# Risk and Distribution of Metastatic Infections by Primary Infection Focus in *Staphylococcus aureus* Bacteremia

**DOI:** 10.1093/ofid/ofaf338

**Published:** 2025-06-25

**Authors:** Seongman Bae, Min Soo Kook, Euijin Chang, Jiwon Jung, Min Jae Kim, Yong Pil Chong, Sung-Han Kim, Sang-Ho Choi, Sang-Oh Lee, Yang Soo Kim

**Affiliations:** Division of Infectious Diseases, Department of Internal Medicine, Asan Medical Center, University of Ulsan College of Medicine, Seoul, Republic of Korea; Center for Antimicrobial Resistance and Microbial Genetics, University of Ulsan College of Medicine, Seoul, Republic of Korea; Division of Infectious Diseases, Department of Internal Medicine, Asan Medical Center, University of Ulsan College of Medicine, Seoul, Republic of Korea; Division of Infectious Diseases, Department of Internal Medicine, Asan Medical Center, University of Ulsan College of Medicine, Seoul, Republic of Korea; Division of Infectious Diseases, Department of Internal Medicine, Asan Medical Center, University of Ulsan College of Medicine, Seoul, Republic of Korea; Division of Infectious Diseases, Department of Internal Medicine, Asan Medical Center, University of Ulsan College of Medicine, Seoul, Republic of Korea; Division of Infectious Diseases, Department of Internal Medicine, Asan Medical Center, University of Ulsan College of Medicine, Seoul, Republic of Korea; Division of Infectious Diseases, Department of Internal Medicine, Asan Medical Center, University of Ulsan College of Medicine, Seoul, Republic of Korea; Division of Infectious Diseases, Department of Internal Medicine, Asan Medical Center, University of Ulsan College of Medicine, Seoul, Republic of Korea; Division of Infectious Diseases, Department of Internal Medicine, Asan Medical Center, University of Ulsan College of Medicine, Seoul, Republic of Korea; Division of Infectious Diseases, Department of Internal Medicine, Asan Medical Center, University of Ulsan College of Medicine, Seoul, Republic of Korea; Center for Antimicrobial Resistance and Microbial Genetics, University of Ulsan College of Medicine, Seoul, Republic of Korea

**Keywords:** endocarditis, metastatic infection, *Staphylococcus aureus*, *Staphylococcus aureus* bacteremia, primary infection focus

## Abstract

**Background:**

*Staphylococcus aureus* bacteremia (SAB) is a significant cause of morbidity and mortality, with a high risk of metastatic infections. Understanding the timing and distribution of metastatic infections based on the primary infection focus is crucial for effective management. We aimed to identify patterns that could guide clinicians in prioritizing surveillance and interventions for patients at high risk of metastatic infection.

**Methods:**

This retrospective cohort study analyzed 1725 patients diagnosed with SAB. We assessed the incidence and distribution of metastatic infections within the cohort, stratifying the data by the timing postdiagnosis and the primary infection focus.

**Results:**

In the cohort of 1725 patients, 289 (16.7%) experienced a total of 439 metastatic infection events within the 90-day follow-up period. The majority of metastatic infections (approximately 85%) occurred within the first 7 days following diagnosis. The incidence of metastatic infections varied significantly with the primary focus of SAB, being highest in patients with endocarditis at 73.4%. The lung was the most frequent metastatic site (23.7%), followed by bones and joints (16.8%) and the central nervous system (12.3%). The distribution of metastatic sites significantly differed according to the primary infection focus.

**Conclusions:**

Our study findings provide essential insights into the risk and distribution of metastatic infections in patients with SAB, highlighting the critical role of the timing and primary infection focus. These findings enable healthcare professionals to adopt a more proactive and targeted approach to managing patients with SAB.

*Staphylococcus aureus* bacteremia (SAB) is associated with significant morbidity and mortality [1, 2]. One of the most clinically challenging aspects of SAB is the risk of metastatic infections, which occur when the pathogen disseminates hematogenously to secondary anatomical sites. These complications are associated with longer hospital stays, increased healthcare costs, and higher mortality rates [3, 4]. Early detection and targeted management of metastatic infections are therefore essential for improving patient outcomes. This principle is reflected in current clinical guidelines for methicillin-resistant *S aureus* (MRSA), which emphasize distinguishing between uncomplicated and complicated SAB. The latter—including cases with metastatic infection—requires prolonged antibiotic therapy, intensive monitoring, and timely source control [5].

Although previous studies have reported that 5%–35% of SAB cases result in metastatic complications [6–8], and have identified certain risk factors such as community acquisition, prolonged bacteremia, or infective endocarditis, few have examined how the primary infection focus influences the risk or anatomical pattern of metastatic spread. Without such data, it remains difficult to apply focused diagnostic strategies or prioritize imaging and specialist consultation based on initial presentation. To address this gap, we conducted a large retrospective cohort study of 1725 adults with SAB over a 10-year period at a tertiary care center. Our goal was to assess the timing, frequency, and distribution of metastatic infections and to determine how these patterns differ depending on the primary site of infection. This information may help clinicians stratify risk more precisely and adopt targeted diagnostic approaches early in the clinical course.

## METHODS

### Study Design, Setting, and Population

This retrospective cohort study included patients who were managed at a 2700-bed tertiary hospital (Asan Medical Center, Seoul, South Korea) for SAB between August 2008 and December 2018. We enrolled nonduplicate patients hospitalized with confirmed SAB, following the exclusion of patients based on predetermined criteria: (*i*) age <18 years; (*ii*) patients discharged from the emergency department; (*iii*) multiple organisms isolated from the same blood sample; (*iv*) a previous history of SAB within 90 days; (*v*) referrals from other hospitals after receiving SAB treatment for 3 or more days; (*vi*) cases of insignificant bacteremia, where *S aureus* was isolated from only 1 blood culture without clinical symptoms of infection, such as fever, and the patient improved without the use of antibiotics against *Staphylococcus*; or (*vii*) cases identified in outpatient clinics.

For all patients, an automatic infectious disease (ID) consultation was triggered at the time of SAB detection, ensuring that each case received standardized evaluation and recommendations by an ID specialist. The *S aureus* research team within the ID department subsequently conducted an initial chart review approximately 1 week after the first positive culture to systematically collect demographic and clinical data, including primary infection foci. A second chart review was performed at 90 days to assess management strategies and clinical outcomes, including mortality, recurrence, and the development of metastatic infections. All patients with SAB received standardized recommendations for evaluating metastatic infections through ID consultations: Blood cultures were followed up at 1- to 2-day intervals until a negative culture result was confirmed, transthoracic echocardiography was performed to screen for endocarditis, and ophthalmological examinations were recommended to check for the presence of endophthalmitis. Furthermore, if new symptoms or findings emerged that were separate from the initial site of infection (such as newly developed back pain or a new pulmonary lesion on chest X-ray), imaging studies such as computed tomography or magnetic resonance imaging of the relevant area were advised. Positron emission tomography (PET) was not routinely recommended.

### Definitions

The primary infection focus for SAB was established as the main source of bacteremia, determined by the presenting symptoms and signs in patients, echocardiographic evaluations, and radiological assessments at the onset of SAB. The infection foci were classified into the following categories: central venous catheter, peripheral venous catheter, pneumonia, skin and soft tissue infection, urinary tract infection, surgical wound infection, endocarditis, bone and joint infection, arteriovenous graft infection, unknown primary focus, and others. Central venous catheter–related bloodstream infections were diagnosed according to the Infectious Diseases Society of America guidelines when either of the following criteria was met: (*i*) a semi-quantitative culture of the removed catheter tip yields >15 colony-forming units by the roll plate method, with the same organism isolated from both the catheter tip and peripheral blood; or (*ii*) a differential time to positivity of >2 hours between catheter and peripheral blood cultures [9]. Moreover, such an infection was also considered if there was at least 1 *S aureus*–positive blood culture in a patient with a catheter, alongside a compatible clinical syndrome and no other discernible infection source. Peripheral venous catheter–related bloodstream infection was established when documented thrombophlebitis at the insertion site of a peripheral intravenous catheter coincided with bacteremia and in the absence of another definitive bacteremia source [10]. The diagnosis of pneumonia required documented pulmonary infiltrates and the isolation of *S aureus* with the same methicillin susceptibility profile (methicillin susceptible [MSSA] or resistant [MRSA]), matching antimicrobial susceptibility patterns, including minimum inhibitory concentrations (MICs), in both blood and respiratory samples [11]. The criteria for infective endocarditis adhered to the modified Duke criteria [12]. Urinary tract infection (UTI) was classified as the primary source of SAB only when *S aureus* was isolated from urine cultures obtained prior to or at the time of SAB diagnosis, in patients with new-onset urinary symptoms (eg, dysuria, frequency, or suprapubic discomfort), to distinguish true UTI from secondary bacteriuria due to hematogenous seeding [13].

Metastatic infection was defined as an *S aureus* infection at an anatomically distinct site from the primary infection focus, identified through clinical symptoms, imaging, or laboratory findings during the course of treatment. The sites of metastatic infection were grouped into the following categories: central nervous system (CNS), heart valve, skin, soft tissue, bone and joint, kidney, eye, lung, and others. These metastatic events were further categorized as early (within 7 days of initial *S aureus* diagnosis) or late (after 7 days). The timing of metastatic infection was defined based on the date the metastatic focus was first identified through imaging, clinical examination, or microbiologic confirmation. This classification did not rely on the reported onset of symptoms, which was often uncertain or unavailable in the retrospective dataset. When multiple foci of infection were present, we classified the primary versus metastatic foci based on a combination of clinical and diagnostic criteria. The timing of detection was the principal determinant—foci identified at initial presentation were preferentially considered as primary. If multiple foci were concurrently identified at initial evaluation, the classification was adjudicated by 2 ID specialists based on a structured review of initial presenting symptoms, the life-threatening potential of each focus, estimated bacterial burden, and the anticipated duration of therapy. Infective endocarditis was categorized as a metastatic focus only when not apparent at initial evaluation and subsequently diagnosed during follow-up imaging prompted by persistent bacteremia or clinical deterioration.

### Ethics

This study was approved by the institutional review board of Asan Medical Center (IRB number: 2013-0234). Given that this study was primarily a retrospective cohort study with structured prospective data collection limited to electronic medical record review, the requirement for informed consent was waived. The waiver was granted because the study did not involve direct patient contact or interventions, all data were obtained from existing medical records, and patient identifiers were encrypted before analysis to ensure confidentiality.

### Statistical Analysis

Categorical variables were analyzed using the χ^2^ test or Fisher exact test, whereas continuous variables were analyzed using Student *t* test. To evaluate potential risk factors for the development of metastatic infection, we performed both univariate and multivariable logistic regression analyses using baseline clinical variables. Variables with a *P* value <.05 in univariate analyses were included in the multivariable model. Odds ratios (ORs) and 95% confidence intervals (CIs) were calculated. The risk of metastatic infection was quantified as the percentage of patients who experienced metastatic events within designated time frames (90 days, within 7 days, and between 7 and 90 days), categorized according to their primary infection focus. To explore the relationship between primary infection foci and the occurrence of metastatic infections, we evaluated the distribution of metastatic infection sites for each primary infection focus. Frequencies and percentages of metastatic infections at various sites were tabulated across different primary infection categories. The χ^2^ test was used to assess the statistical significance of the observed distributions. To examine the association between site-specific metastatic infections and 90-day mortality and recurrence, multivariable logistic regression analyses were conducted. These analyses adjusted for age, sex, comorbidities, the severity of infection, and the primary infection focus of SAB. All reported *P* values were 2-sided, and a *P* value <.05 was considered statistically significant. Data manipulation and statistical analyses were performed using R, version 4.0.4 (R Foundation for Statistical Computing, Vienna, Austria).

## RESULTS

**Figure 1.**
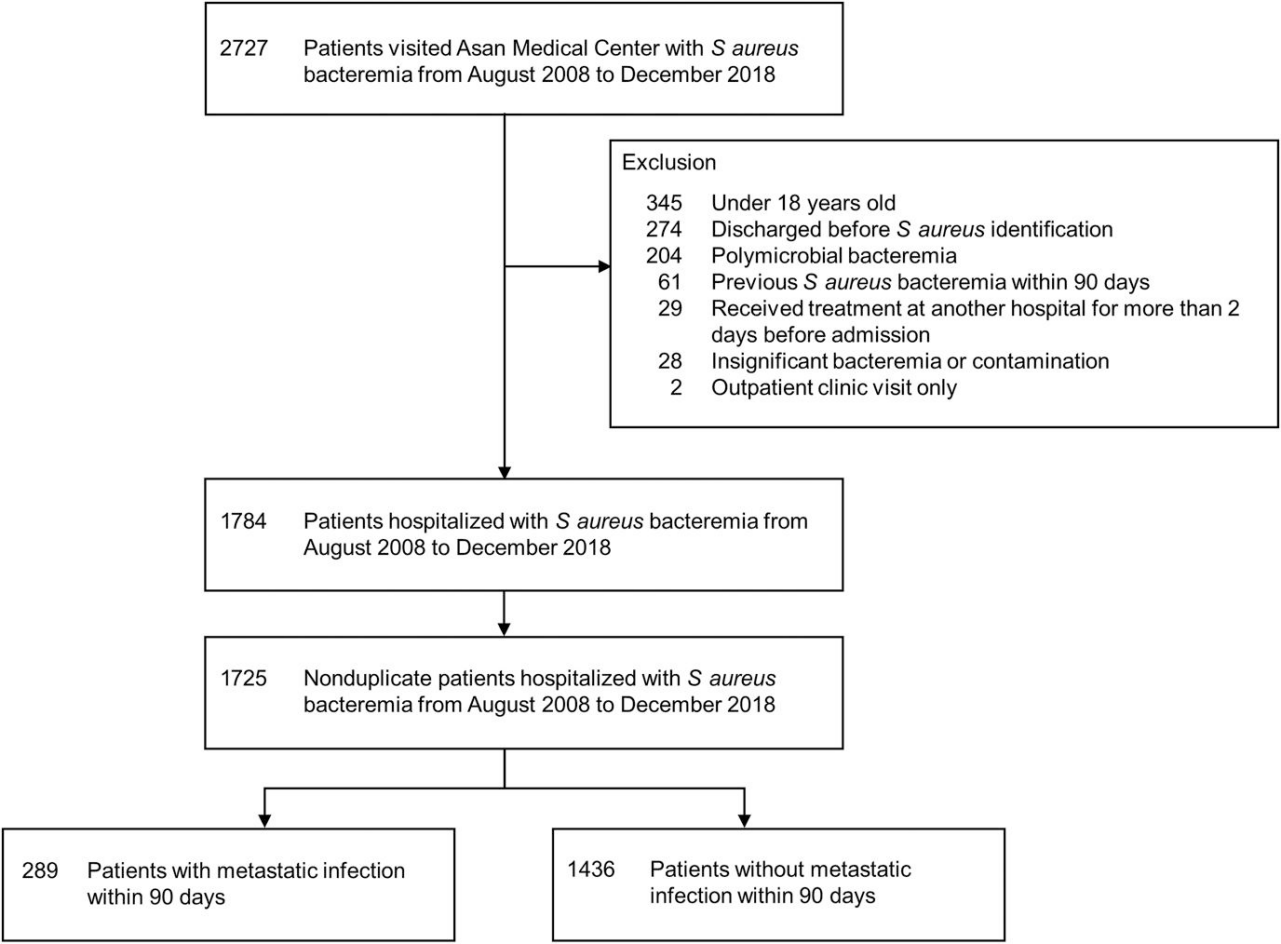
Study inclusion and exclusion flowchart.

A total of 1725 patients were included in this study (Figure 1): 1190 (69.0%) completed the 90-day follow-up, 475 (27.5%) died before reaching 90 days, and 61 (3.5%) were lost to follow-up. The median age was 63 years, 55.4% were male, and 51.3% had infections caused by MRSA (Table 1). Echocardiography was performed on 82.3% of patients, while ophthalmologic examinations were conducted on 66.1% (Supplementary Table 1). Among the study population, 16.8% (289/1725) experienced at least 1 metastatic infection during the 90-day period; of these, 80.3% (232/289) experienced a metastatic infection within 7 days, 11.4% (33/289) after 7 days, and 8.3% (24/289) during both periods (Supplementary Table 2).

**Table 1.** Baseline Characteristics of the Patients With *Staphylococcus aureus* Bacteremia

Characteristic	Metastatic Infection (n = 289)	No Metastatic Infection (n = 1436)	Total (n = 1725)	*P* Value
Age, y, median (IQR)	63 (51–70)	63 (53–71.5)	63 (52–71)	.568
Age >60 y	161 (55.7)	795 (55.4)	956 (55.4)	.965
Male sex	891 (62.0)	183 (63.3)	1074 (62.3)	.733
Mode of acquisition				<.001
Community-acquired	82 (28.4)	162 (11.3)	244 (14.1)	
Healthcare-associated	91 (31.5)	423 (29.5)	514 (29.8)	
Nosocomial	116 (40.1)	851 (59.3)	967 (56.1)	
MRSA	139 (48.1)	746 (51.9)	885 (51.3)	.258
Underlying medical conditions				
Malignancy	106 (36.7)	701 (48.8)	807 (46.8)	<.001
Diabetes	88 (30.4)	447 (31.1)	535 (31.0)	.875
Hypertension	111 (38.4)	591 (41.2)	702 (40.7)	.423
End-stage renal disease	37 (12.8)	151 (10.5)	188 (10.9)	.301
Liver cirrhosis	39 (13.5)	228 (15.9)	267 (15.5)	.351
Immunosuppressant agent	16 (5.5)	100 (7.0)	116 (6.7)	.450
Corticosteroid use	54 (18.7)	383 (26.7)	437 (25.3)	.006
CCI score, median (IQR)	2 (1–4)	3 (2–5)	3 (2–5)	<.001
Indwelling prosthetic devices				
Central venous catheter	94 (32.5)	590 (41.1)	684 (39.7)	.008
Cardiac implantable device	3 (1.0)	19 (1.3)	22 (1.3)	.915
Prosthetic heart valve	18 (6.2)	42 (2.9)	60 (3.5)	.009
Vascular graft	28 (9.7)	105 (7.3)	133 (7.7)	.207
Orthopedic implant	17 (5.9)	51 (3.6)	68 (3.9)	.091
Bacteremia characteristics				
Fever	205 (70.9)	937 (65.3)	1142 (66.2)	.073
Fever >72 h	118 (40.8)	351 (24.4)	469 (27.2)	<.001
Severity of infection				.013
No sepsis	48 (16.6)	273 (19.0)	321 (18.6)	
Sepsis	195 (67.5)	1019 (71.0)	1214 (70.4)	
Septic shock	46 (15.9)	144 (10.0)	190 (11.0)	
Primary infection focus				
Central venous catheter	73 (25.3)	386 (26.9)	459 (26.6)	.620
Peripheral venous catheter	11 (3.8)	108 (7.5)	119 (6.9)	.032
Pneumonia	4 (1.4)	146 (10.2)	150 (8.7)	<.001
Skin and soft tissue infection	31 (10.7)	135 (9.4)	166 (9.6)	.557
Urinary tract infection	1 (0.3)	22 (1.5)	23 (1.3)	.186
Surgical wound infection	13 (4.5)	85 (5.9)	98 (5.7)	.416
Endocarditis^a^	47 (16.3)	17 (1.2)	64 (3.7)	<.001
Bone	28 (9.7)	70 (4.9)	98 (5.7)	.002
Joint	14 (4.8)	43 (3.0)	57 (3.3)	.15
Arteriovenous graft infection	16 (5.5)	32 (2.2)	48 (2.8)	.003
Others^b^	15 (5.2)	158 (11.0)	173 (10.0)	.004
Unknown primary focus	36 (12.5)	234 (16.3)	270 (15.7)	.121
C-reactive protein ≥10 mg/dL	195 (67.5)	607 (42.3)	802 (46.5)	<.001

Data are presented as No. (%) unless otherwise indicated. *P* values correspond to comparisons between patients with and without metastatic infection.

Abbreviations: CCI, Charlson Comorbidity Index; IQR, interquartile range; MRSA, methicillin-resistant *Staphylococcus aureus*.

^a^Among patients with endocarditis, all 17 without metastatic infection had left-sided infective endocarditis (IE). Among the 47 with metastatic infection, 40 (85.1%) had left-sided IE, 6 (12.8%) right-sided IE, and 1 (2.1%) had both-sided involvement.

^b^Others included intra-abdominal infection, parotitis, and central nervous system infection.

### Risk Factors Associated With Metastatic Infection

To examine factors potentially associated with metastatic infection, logistic regression analyses were performed using baseline clinical variables. In univariate logistic regression analysis, nosocomial acquisition, malignancy, corticosteroid use, central venous catheter, prosthetic heart valve, persistent fever (>72 hours), prolonged bacteremia (≥3 days), septic shock, pneumonia, endocarditis, bone infection, arteriovenous graft infection, C-reactive protein (CRP) ≥10 mg/dL, and prolonged antibiotic treatment (>6 weeks) were significantly associated with metastatic infection (Table 2 and Supplementary Table 3). In the multivariable analysis including variables with univariate *P* < .05, independent risk factors for metastatic infection included nosocomial acquisition (OR, 0.50 [95% CI, .31–.80]), persistent fever (OR, 1.54 [95% CI, 1.12–2.11]), prolonged bacteremia (OR, 3.74 [95% CI, 2.77–5.09]), septic shock (OR, 1.72 [95% CI, 1.00–2.97]), pneumonia (OR, 0.15 [95% CI, .04–.37]), infective endocarditis (OR, 10.00 [95% CI, 5.19–20.00]), arteriovenous graft infection (OR, 2.73 [95% CI, 1.28–5.68]), CRP ≥10 mg/dL (OR, 1.97 [95% CI, 1.44–2.70]), and antibiotic duration >6 weeks (OR, 1.91 [95% CI, 1.19–3.13]) (Table 2).

**Table 2.** Univariate and Multivariable Logistic Regression Analyses of Factors Associated With Metastatic Infection

Variable	Univariate			Multivariable		
	OR	(95% CI)	*P* Value	OR	(95% CI)	*P* Value
Mode of acquisition						
Community-acquired	(reference)			(reference)		
Healthcare-associated	0.42	(.30–.60)	<.001	0.58	(.37–.91)	.02
Nosocomial	0.27	(.19–.38)	<.001	0.5	(.31–.80)	.004
Malignancy	0.61	(.47–.79)	<.001	1.14	(.83–1.57)	.43
Corticosteroid use	0.63	(.46–.86)	.01	0.87	(.60–1.25)	.47
Central venous catheter	0.69	(.53–.90)	.01	1.05	(.72–1.52)	.81
Prosthetic heart valve	2.20	(1.22–3.83)	.01	1.03	(.47–2.13)	.94
Fever >72 h	2.13	(1.64–2.77)	<.001	1.54	(1.12–2.11)	.01
Persistent bacteremia (≥3 d)	5.21	(3.98–6.85)	<.001	3.74	(2.77–5.09)	<.001
Severity of infection						
No sepsis	(reference)			(reference)		
Sepsis	1.09	(.78–1.55)	.63	0.81	(.54–1.21)	.29
Septic shock	1.82	(1.15–2.86)	.01	1.72	(1.00–2.97)	.05
Peripheral venous catheter	0.49	(.24–.88)	.03	1.07	(.49–2.14)	.86
Pneumonia	0.12	(.04–.30)	<.001	0.15	(.04–.37)	<.001
Endocarditis	16.20	(9.34–29.50)	<.001	10.00	(5.19–20.00)	<.001
Bone infection	2.09	(1.31–3.27)	.002	0.98	(.55–1.70)	.94
Arteriovenous graft infection	2.57	(1.36–4.68)	.003	2.73	(1.28–5.68)	.01
Others	0.44	(.25–.74)	.003	0.61	(.32–1.08)	.10
C-reactive protein ≥10 mg/dL	2.83	(2.17–3.71)	<.001	1.97	(1.44–2.70)	<.001
Duration of antibiotic therapy						
0–2 wk	(reference)			(reference)		
>2–6 wk	1.09	(.74–1.65)	.68	0.94	(.61–1.48)	.78
>6 wk	3.76	(2.50–5.79)	<.001	1.91	(1.19–3.13)	.01

Abbreviations: CI, confidence interval; OR, odds ratio.

### 90-Day Metastatic Infections by Primary Infection Focus of SAB

**Figure 2.**
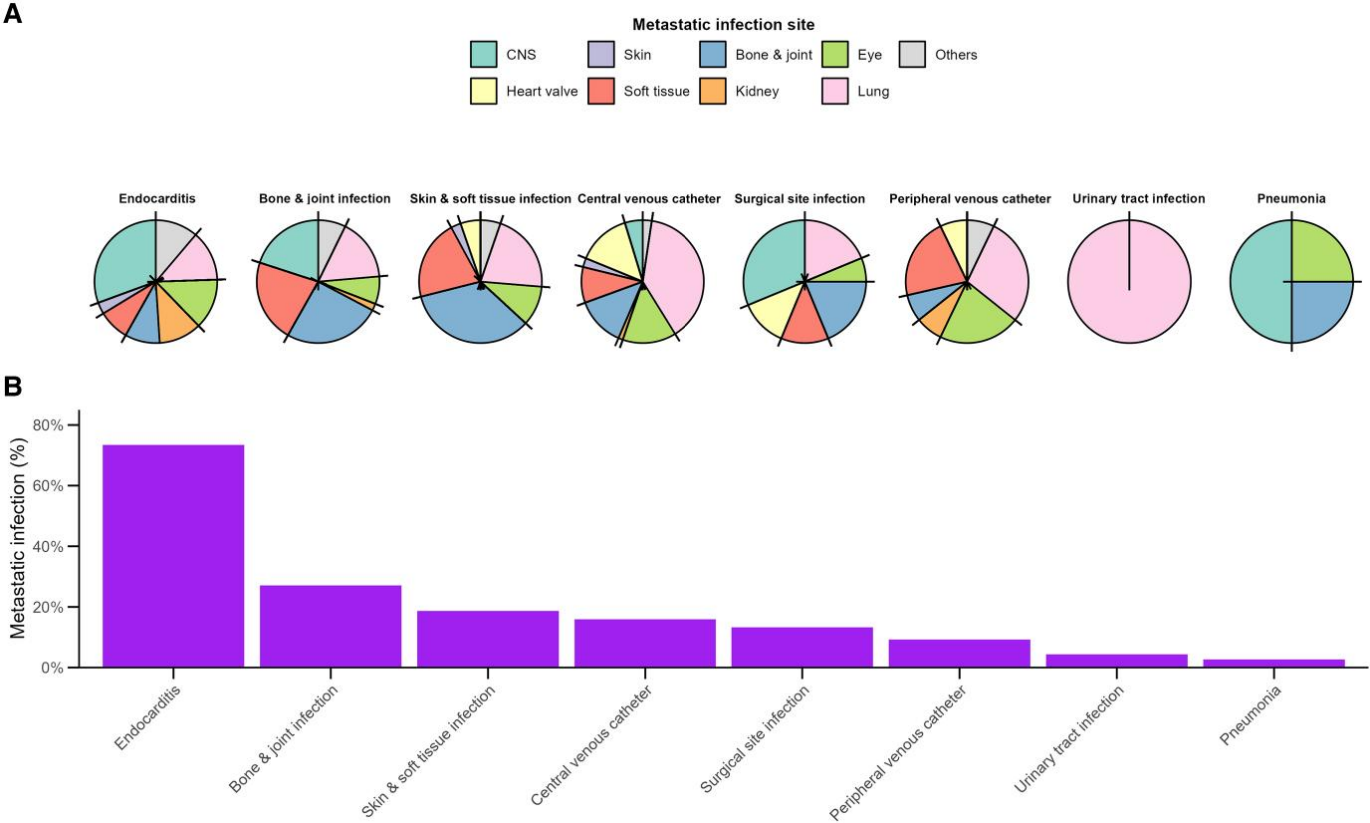
Distribution of metastatic infections by primary infection focus in *Staphylococcus aureus* bacteremia. *A*, Risk of metastatic infections according to primary infection foci in *S aureus* bacteremia patients. *B*, Pie charts depicting the proportional distributions of metastatic infection sites, organized by primary infection focus in descending order of metastatic infection risk. Abbreviation: CNS, central nervous system.

**Figure 3.**
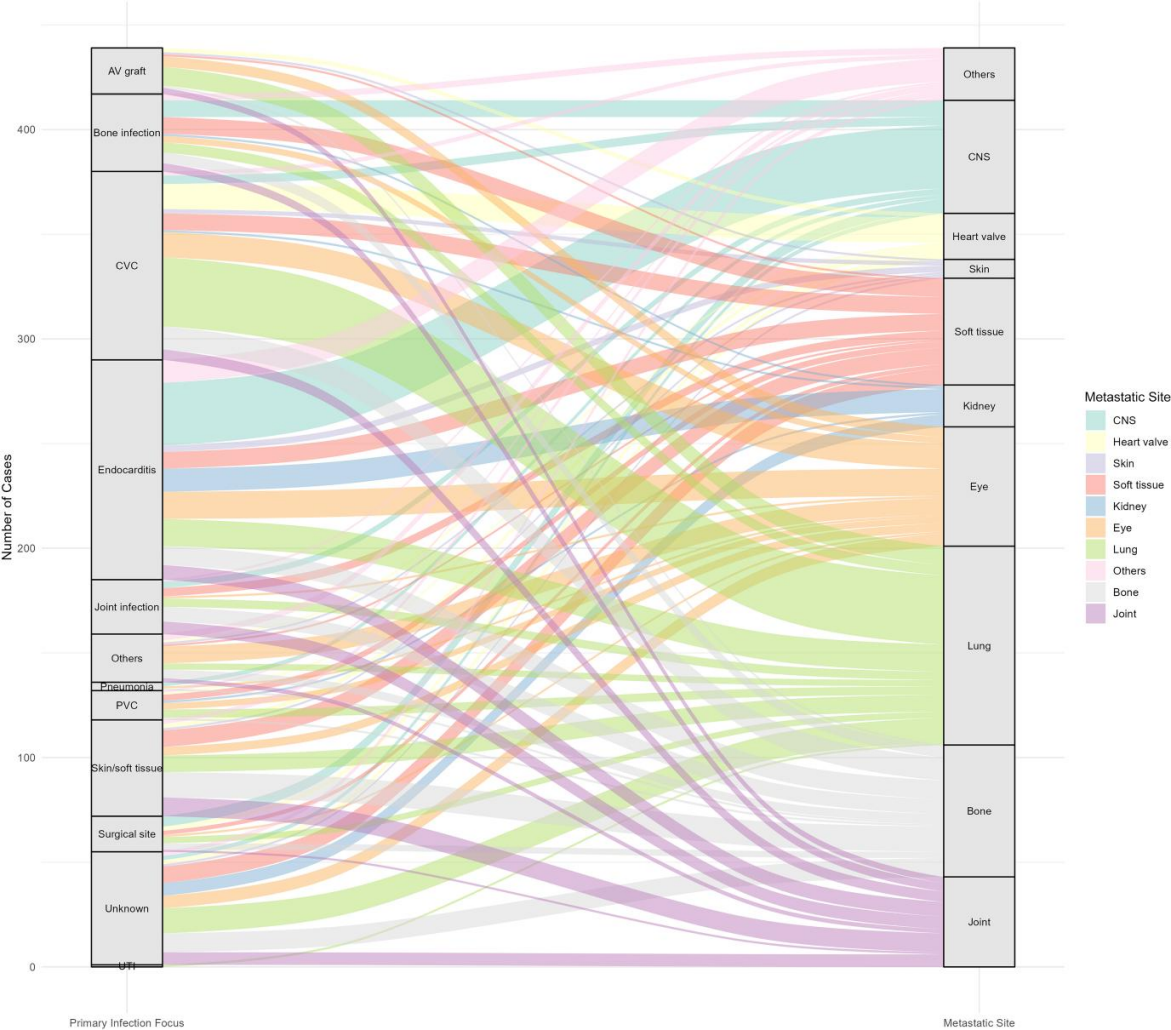
Alluvial plot illustrating the distribution of metastatic infection sites by primary infection focus. Each flow represents the number of patients who developed a specific metastatic infection type from each primary focus. Abbreviations: AV, arteriovenous; CNS, central nervous system; CVC, central venous catheter; PVC, peripheral venous catheter.

During the 90-day period, a total of 439 metastatic infection events were identified among these 289 patients, and significant variation in the incidence of metastatic infection was observed according to the infection focus of SAB (Figure 2*B*). In terms of infection foci associated with SAB, the incidence was highest for endocarditis at 73.4%, followed by infections in arteriovenous grafts (33.3%), bone infections (28.6%), and joint infections (24.6%)—all representing metastatic infection rates exceeding 20% (Supplementary Table 2). The lung was the most frequent site among the 400 metastatic infection events, accounting for 23.7% (104/439), followed by eye (13.0% [57/439]), CNS (12.3% [54/439]), bone (12.3% [54/439]), soft tissue (11.6% [51/439]), and joint (9.8% [43/439]) (Supplementary Table 4). The distribution of metastatic infection sites exhibited significant variance according to the infection focus (Figure 2*A* and Figure 3): The proportions of metastatic infections to the CNS, kidney, eye, lung, and other sites differed statistically based on the primary infection site (Supplementary Table 4). Among patients with eye involvement (n = 57), 22 (38.6%) reported ocular symptoms such as decreased visual acuity, visual disturbance, or eye pain. In contrast, 23 patients (40.4%) were asymptomatic and diagnosed based on screening ophthalmologic evaluations. In the remaining 12 patients (21.1%), symptom assessment was not possible due to factors such as altered mental status or mechanical ventilation (Supplementary Table 5).

### Early and Late Metastatic Infections by Infection Focus

Among the 439 metastatic infection episodes identified within 90 days of SAB onset, 363 (82.7%) were recognized within the first 7 days and 76 (17.3%) thereafter. The incidence and distribution of both early and late metastatic infections varied by the primary infection focus. Early metastatic infections were most frequently associated with endocarditis (73.4%), followed by arteriovenous graft infections (29.2%) and bone infection (23.5%) and joint infections (21.1%) (Supplementary Table 2). In contrast, late metastatic infections were also most commonly linked to endocarditis (14.1%), with joint infections (10.5%) and arteriovenous graft infections (6.2%) following. Regarding the anatomical sites involved, early infections most commonly affected the lungs (24.7%), CNS (13.9%), soft tissue (13.3%), and eye (12.8%) (Supplementary Table 6 and Supplementary Figure 1). For late infections, bone and joint involvement was predominant (57.6%), followed by the eye (15.1%), lung (8.2%), heart valves (6.8%), and CNS (5.5%) (Supplementary Table 7 and Supplementary Figure 2).

### Metastatic Infections in Patients With MSSA and MRSA Bacteremia

In the MSSA subgroup (n = 227), the most frequently observed metastatic infection sites were the lung (17.6%), eye (15.4%), and bone (14.1%), followed by CNS (13.7%) and soft tissue (11.9%) (Supplementary Table 8). In the MRSA subgroup (n = 212), the lung (25.9%) was the most common site, followed by bone (14.6%), soft tissue (11.3%), joint (11.3%), and CNS (10.8%) (Supplementary Table 9). Among metastatic sites, only CNS involvement showed a statistically significant difference across primary infection sites in the MSSA group (*P* < .001). In contrast, in the MRSA group, CNS (*P* < .001), bone (*P* = .047), kidney (*P* < .001), and eye (*P* = .023) involvements varied significantly according to the primary infection site.

### Association Between Metastatic Infections and Clinical Outcomes

**Figure 4.**
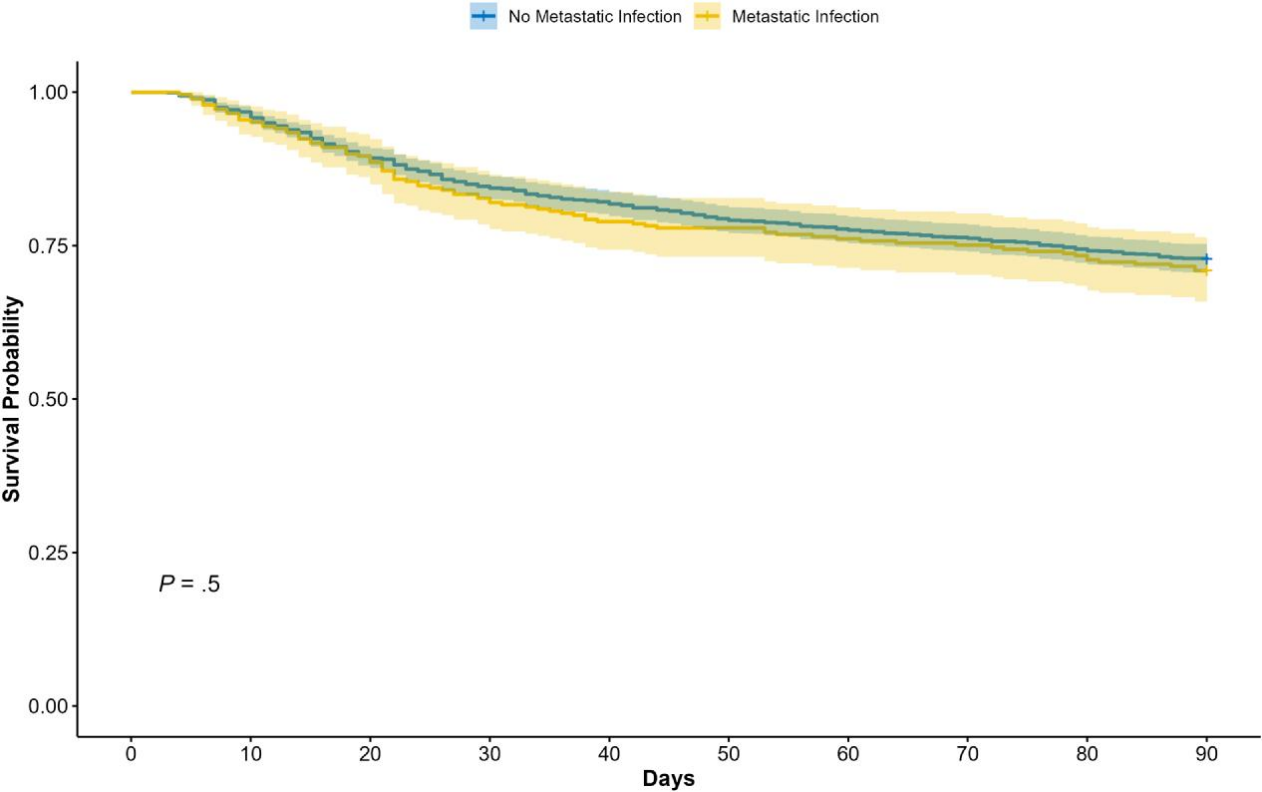
Kaplan-Meier survival curves comparing 90-day mortality between patients with and without metastatic infection. Survival probabilities were not significantly different between groups (log-rank *P* = .5).

Patients with metastatic infections underwent significantly longer courses of antibiotic treatment than those without metastatic infections. The crude all-cause mortality rates (29.1% vs 27.2%) and recurrence rates (5.2% vs 4.9%) within 90 days were not significantly different between patients with versus without metastatic infections (Supplementary Table 1). Kaplan-Meier survival analysis also showed no significant difference in 90-day survival between the 2 groups (log-rank *P* = .5, Figure 4). In the multivariable logistic regression analysis adjusted for age, sex, comorbidities, septic shock, and the primary focus of SAB, a significant association was observed between 90-day mortality and metastatic infections involving the CNS, heart valves, soft tissue, and lungs (Supplementary Table 10). No significant association was found between the site of metastatic infection and 90-day recurrence (Supplementary Table 10).

## DISCUSSION

This study has provided a comprehensive analysis of the risk and distribution of metastatic sites in a large cohort of patients with SAB, focusing particularly on the timing and primary infection site. Our findings demonstrate that the risk of metastatic infections significantly varies depending on the primary infection site, with conditions like endocarditis being more prone to metastatic spread. There was a notable variation in the distribution of metastatic sites according to the primary focus of SAB. Specifically, metastatic infections involving the CNS, heart valves, and lungs were linked to increased 90-day mortality and showed distinct variations based on the initial site of infection. Importantly, only about 40% of patients with ocular involvement reported symptoms at presentation, while the remainder were either asymptomatic or unable to report symptoms, underscoring the need for routine ophthalmologic screening in all SAB cases, regardless of visual complaints. These insights allow clinicians to adopt a proactive approach to managing SAB by identifying potential metastatic sites based on the primary infection focus.

During the 90-day observation period, 16.8% of patients in our study developed metastatic infections, aligning with the 5%–35% range reported in previous studies [6–8, 14]. Notably, studies incorporating routine PET scans have documented metastatic infection rates exceeding 70% [15, 16]. Therefore, the relatively lower metastatic infection rate observed in our study may be attributable to our methodology, which did not routinely employ PET scans and relied primarily on new signs or symptoms to guide further imaging. However, the routine use of PET scans has not been conclusively associated with improved mortality outcomes in SAB patients [17]. Despite the absence of demonstrated survival benefits from PET scans, proactive measures for detecting metastatic infections—such as routine transthoracic echocardiography and ophthalmological examinations, along with symptom-based, site-specific computed tomography or magnetic resonance imaging—are crucial in enhancing the management of SAB. Our data underscore the importance of these interventions, particularly given the significant association between certain metastatic sites (CNS, heart valves, and lungs) and 90-day mortality.

In this study, we included endocarditis and hematogenous bone and joint infections as primary infection sites of SAB, in addition to the commonly considered portals of entry such as catheters, the skin and soft tissue, surgical sites, and pneumonia. Endocarditis and hematogenous bone and joint infections are often regarded as metastatic infection sites secondary to an unknown focus of infection due to their ambiguous portals of entry, thus are conventionally classified as “unknown primary bacteremia” [18]. However, distinguishing between unknown and specific sites like endocarditis and bone and joint infection in this study is justified for several reasons: First, significant differences in initial symptoms, clinical course, and prognosis exist between SAB from unknown origins and that associated with endocarditis or bone and joint infection [19–21]. Second, endocarditis and bone and joint infections are not merely the final stages of metastatic infection; they frequently accompany secondary and tertiary metastatic infections to other sites, as our results demonstrate. Third, multiple clinical trials, such as the Combination Antibiotics for Methicillin-Resistant Staphylococcus aureus-2 and Adjunctive Rifampicin for Staphylococcus aureus Bacteraemia trials, have categorized endocarditis and bone and joint infections as distinct foci or sources of infection, supporting their separation for a more relevant clinical approach [22, 23]. This approach of differentiating between unspecified primary sites and specific infections like endocarditis or bone and joint infections aims to offer healthcare providers a more effective methodology for the diagnosis and early management of SAB.

There were several limitations to this study. First, as a single-center observational study, its results may have limited generalizability. Second, this study had a high MRSA rate (approximately 50%), and different outcomes might be observed in locales with lower MRSA prevalence. Third, this study lacked data on the profiles of virulence genes or differentially expressed genes among *S aureus* isolates causing metastatic infections. Therefore, further research incorporating microbiological variables for metastatic infections in SAB is necessary. Fourth, the classification of metastatic infections as early versus late was based on the timing of detection, not symptom onset, which was often unavailable in this retrospective cohort. This approach may not fully capture the true onset or biological progression of metastatic spread and reflects the limitations inherent in retrospective identification. Fifth, due to the retrospective nature of this study, there is an inherent risk of misclassification. In particular, the distinction between primary and metastatic infection foci may have been biased by the timing of diagnosis or variable documentation in medical records. Furthermore, certain metastatic sites may have remained undetected or were identified late, especially in cases without routine advanced imaging such as PET scans. These limitations should be considered when interpreting the reported risk profiles.

In conclusion, this study's findings offer a detailed examination of the risk factors and distribution of metastatic infections associated with SAB. By stratifying patients according to their primary infection foci, clinicians can adopt a more informed and proactive approach to management, potentially tailoring surveillance and intervention strategies to mitigate SAB-associated risk.

## Supplementary Material

ofaf338_Supplementary_Data

## Supplementary Data

Supplementary materials are available at *Open Forum Infectious Diseases* online. Consisting of data provided by the authors to benefit the reader, the posted materials are not copyedited and are the sole responsibility of the authors, so questions or comments should be addressed to the corresponding author.

## Notes

***Disclaimer.*** The funders had no role in the study design, data collection and analysis, decision to publish, or preparation of the manuscript.

***Financial support.*** This research was supported by the Korea National Institute of Health research project (grant number 2022ER200500 to Y. S. K.) and the Asan Institute for Life Sciences, Asan Medical Center, Seoul, Korea (grant number 2024IF0018-1 to S. B.).

## References

[1] GBD 2019 Antimicrobial Resistance Collaborators . Global mortality associated with 33 bacterial pathogens in 2019: a systematic analysis for the Global Burden of Disease Study 2019. Lancet 2022; 400:2221–48. 36423648 10.1016/S0140-6736(22)02185-7 PMC9763654

[2] Tom S, Galbraith JC, Valiquette L, et al Case fatality ratio and mortality rate trends of community-onset *Staphylococcus aureus* bacteraemia. Clin Microbiol Infect 2014; 20:O630–2. 24461038 10.1111/1469-0691.12564

[3] Fowler VG Jr, Olsen MK, Corey GR, et al Clinical identifiers of complicated *Staphylococcus aureus* bacteremia. Arch Intern Med 2003; 163:2066–72. 14504120 10.1001/archinte.163.17.2066

[4] Kinamon T, Dagher M, Park L, Ruffin F, Fowler VG Jr, Maskarinec SA. Risk factors and outcomes of hematogenous vertebral osteomyelitis in patients with *Staphylococcus aureus* bacteremia. Clin Infect Dis 2023; 77:1226–33. 37747828 10.1093/cid/ciad377 PMC10640688

[5] Liu C, Bayer A, Cosgrove SE, et al Clinical practice guidelines by the Infectious Diseases Society of America for the treatment of methicillin-resistant *Staphylococcus aureus* infections in adults and children. Clin Infect Dis 2011; 52:e18–55. 21208910 10.1093/cid/ciq146

[6] Lesens O, Hansmann Y, Brannigan E, et al Positive surveillance blood culture is a predictive factor for secondary metastatic infection in patients with *Staphylococcus aureus* bacteraemia. J Infect 2004; 48:245–52. 15001303 10.1016/j.jinf.2003.10.010

[7] Sullivan SB, Austin ED, Stump S, et al Reduced vancomycin susceptibility of methicillin-susceptible *Staphylococcus aureus* has no significant impact on mortality but results in an increase in complicated infection. Antimicrob Agents Chemother 2017; 61:e00316-17. 28507105 10.1128/AAC.00316-17 PMC5487643

[8] Horino T, Hori S. Metastatic infection during *Staphylococcus aureus* bacteremia. J Infect Chemother 2020; 26:162–9. 31676266 10.1016/j.jiac.2019.10.003

[9] Mermel LA, Allon M, Bouza E, et al Clinical practice guidelines for the diagnosis and management of intravascular catheter-related infection: 2009 update by the Infectious Diseases Society of America. Clin Infect Dis 2009; 49:1–45. 19489710 10.1086/599376 PMC4039170

[10] Blauw M, Foxman B, Wu J, Rey J, Kothari N, Malani AN. Risk factors and outcomes associated with hospital-onset peripheral intravenous catheter–associated *Staphylococcus aureus* bacteremia. Open Forum Infect Dis 2019; 6:ofz111. 30949543 10.1093/ofid/ofz111 PMC6441569

[11] González C, Rubio M, Romero-Vivas J, González M, Picazo JJ. Bacteremic pneumonia due to *Staphylococcus aureus*: a comparison of disease caused by methicillin-resistant and methicillin-susceptible organisms. Clin Infect Dis 1999; 29:1171–7. 10524959 10.1086/313440

[12] Li JS, Sexton DJ, Mick N, et al Proposed modifications to the Duke criteria for the diagnosis of infective endocarditis. Clin Infect Dis 2000; 30:633–8. 10770721 10.1086/313753

[13] Schuler F, Barth PJ, Niemann S, Schaumburg F. A narrative review on the role of *Staphylococcus aureus* bacteriuria in *S. aureus* bacteremia. Open Forum Infect Dis 2021; 8:ofab158. 34189162 10.1093/ofid/ofab158 PMC8233567

[14] Fowler VG Jr, Sanders LL, Sexton DJ, et al Outcome of *Staphylococcus aureus* bacteremia according to compliance with recommendations of infectious diseases specialists: experience with 244 patients. Clin Infect Dis 1998; 27:478–86. 9770144 10.1086/514686

[15] Berrevoets MAH, Kouijzer IJE, Aarntzen E, et al (18)F-FDG PET/CT optimizes treatment in *Staphylococcus aureus* bacteremia and is associated with reduced mortality. J Nucl Med 2017; 58:1504–10. 28336786 10.2967/jnumed.117.191981

[16] Ghanem-Zoubi N, Kagna O, Abu-Elhija J, et al Integration of FDG-PET/CT in the diagnostic workup for *Staphylococcus aureus* bacteremia: a prospective interventional matched-cohort study. Clin Infect Dis 2021; 73:e3859–66. 32639560 10.1093/cid/ciaa929

[17] van der Vaart TW, Prins JM, van Werkhoven CH, et al Positive impact of [18F]FDG-PET/CT on mortality in patients with *Staphylococcus aureus* bacteremia explained by immortal time bias. Clin Infect Dis 2023; 77:9–15. 36869816 10.1093/cid/ciad112 PMC10320066

[18] del Rio A, Cervera C, Moreno A, Moreillon P, Miró JM. Patients at risk of complications of *Staphylococcus aureus* bloodstream infection. Clin Infect Dis 2009; 48(Suppl 4):S246–53. 19374580 10.1086/598187

[19] Miro JM, Anguera I, Cabell CH, et al *Staphylococcus aureus* native valve infective endocarditis: report of 566 episodes from the International Collaboration on Endocarditis merged database. Clin Infect Dis 2005; 41:507–14. 16028160 10.1086/431979

[20] Bassetti M, Peghin M, Trecarichi EM, et al Characteristics of *Staphylococcus aureus* bacteraemia and predictors of early and late mortality. PLoS One 2017; 12:e0170236. 28152067 10.1371/journal.pone.0170236 PMC5289427

[21] van Hal SJ, Jensen SO, Vaska VL, Espedido BA, Paterson DL, Gosbell IB. Predictors of mortality in *Staphylococcus aureus* bacteremia. Clin Microbiol Rev 2012; 25:362–86. 22491776 10.1128/CMR.05022-11 PMC3346297

[22] Tong SY, Nelson J, Paterson DL, et al CAMERA2—combination antibiotic therapy for methicillin-resistant *Staphylococcus aureus* infection: study protocol for a randomised controlled trial. Trials 2016; 17:170. 27029920 10.1186/s13063-016-1295-3 PMC4815121

[23] Thwaites GE, Scarborough M, Szubert A, et al Adjunctive rifampicin for *Staphylococcus aureus* bacteraemia (ARREST): a multicentre, randomised, double-blind, placebo-controlled trial. Lancet 2018; 391:668–78. 29249276 10.1016/S0140-6736(17)32456-X PMC5820409

