# Supplementary material for: Risk and Distribution of Metastatic Infections by Primary Infection Focus in Staphylococcus aureus Bacteremia
Source: Open Forum Infect Dis. 2025 Jun 25;12(6):ofaf338. doi: 10.1093/ofid/ofaf338 (PMC12188215; doi:10.1093/ofid/ofaf338)
Supplement: ofaf338_Supplementary_Data [file ofaf338_supplementary_data.docx]

**Table of Contents**

| **Supplementary Table 1. Management and treatment outcomes in patients with *Staphylococcus aureus* bacteremia** | Page 2 |
| --- | --- |
| **Supplementary Table 2. 90-day metastatic infection risk according to infection focus of *Staphylococcus aureus* bacteremia.** | Page 3 |
| **Supplementary Table 3. Univariate logistic regression analysis of risk factors associated with metastatic infection in patients with *S. aureus* bacteremia** | Page 4 |
| **Supplementary Table 4. Distribution of metastatic infection sites by primary infection focus within 90 days post–*Staphylococcus aureus* bacteremia** | Page 6 |
| **Supplementary Table 5. Detailed symptom profile of ocular involvement in patients with *S. aureus* bacteremia** | Page 7 |
| **Supplementary Table 6. Distribution of early metastatic infection sites (within 7 days post-bacteremia) by primary infection focus** | Page 8 |
| **Supplementary Table 7. Distribution of late metastatic infection sites (beyond 7 days post-bacteremia) by primary infection focus** | Page 9 |
| **Supplementary Table 8. Metastatic infection site distribution in patients with MSSA bacteremia** | Page 10 |
| **Supplementary Table 9. Metastatic infection site distribution in patients with MRSA bacteremia** | Page 11 |
| **Supplementary Table 10. Multivariable logistic regression analysis for the association between clinical outcomes and metastatic infections** | Page 12 |
| **Supplementary Figure 1. Early metastatic infection profiles by primary infection focus in *Staphylococcus aureus* bacteremia.** | Page 13 |
| **Supplementary Figure 2. Late metastatic infection profiles by primary infection focus in *Staphylococcus aureus* bacteremia.** | Page 14 |

**Supplementary Table 1. Management and treatment outcomes in patients with *Staphylococcus aureus* bacteremia**

|  | **Metastatic infection (n=289)** | **No metastatic infection (n=1,436)** | **Total (n=1,725)** | ***P* value** |
| --- | --- | --- | --- | --- |
| Echocardiography | 277 (95.8) | 1,152 (80.2) | 1,429 (82.8) | <.001 |
| Transthoracic | 276 (95.5) | 1,144 (79.7) | 1,420 (82.3) | <.001 |
| Transesophageal | 120 (41.5) | 134 (9.3) | 254 (14.7) | <.001 |
| Ophthalmological exam | 240 (83.0) | 900 (62.7) | 1,140 (66.1) | <.001 |
| Time to appropriate antibiotic therapy, d | 1 ( 0–1) | 0 ( 0–1.5) | 0 ( 0–1) | .495 |
| Duration of antibiotic therapy, d | 35 (20–56) | 21 (15–33) | 22 (15–37) | <.001 |
| Focus removal |  |  |  | .585 |
| Complete | 128 (44.3) | 674 (46.9) | 802 (46.5) |  |
| Not complete | 143 (49.5) | 689 (48.0) | 832 (48.2) |  |
| No eradicable focus | 18 (6.2) | 73 (5.1) | 91 (5.3) |  |
| Duration of bacteremia, d | 4 ( 1–9) | 1 ( 1–3) | 1 ( 1–4) | <.001 |
| Persistent bacteremia (≥3 d) | 193 (66.8) | 400 (27.9) | 593 (34.4) | <.001 |
| 90-day outcomes |  |  |  |  |
| Death | 84 (29.1) | 391 (27.2) | 475 (27.5) | .572 |
| Recurrence | 15 (5.2) | 70 (4.9) | 85 (4.9) | .938 |
| Length of hospital stay, d | 33 (20–56) | 23 (14–42) | 24 (15–44) | <.001 |

Data are presented as n (%) or medians (interquartile range). *P* values correspond to comparisons between patients with and without metastatic infection.

**Supplementary Table 2. 90-day metastatic infection risk according to infection focus of *Staphylococcus aureus* bacteremia**

|  | **CVC**  **(n=459)** | **PVC**  **(n=119)** | **Lung**  **(n=150)** | **SSTI**  **(n=166)** | **UTI**  **(n=23)** | **SSI**  **(n=98)** | **IE**  **(n=64)** | **Bone**  **(n=98)** | **Joint**  **(n=57)** | **AVG infection**  **(n=48)** | **Others**  **(n=173)** | **Unknown**  **(n=270)** | **Total**  **(n=1725)** | ***P* Value** |
| --- | --- | --- | --- | --- | --- | --- | --- | --- | --- | --- | --- | --- | --- | --- |
| During 90 d | 73 (15.9) | 11 (9.2) | 4 (2.7) | 31 (18.7) | 1 (4.3) | 13 (13.3) | 47 (73.4) | 28 (28.6) | 14 (24.6) | 16 (33.3) | 15 (8.7) | 36 (13.3) | 289 (16.8) | <.001 |
| Early (≤7 d) | 68 (14.8) | 10 (8.4) | 3 (2.0) | 25 (15.1) | 1 (4.3) | 11 (11.2) | 47 (73.4) | 23 (23.5) | 12 (21.1) | 14 (29.2) | 14 (8.1) | 28 (10.4) | 256 (14.8) | <.001 |
| Late (>7 d) | 8 (1.7) | 2 (1.7) | 1 (0.7) | 8 (4.8) | 0 (0) | 3 (3.1) | 9 (14.1) | 5 ( 5.1) | 6 (10.5) | 3 (6.2) | 1 (0.6) | 11 (4.1) | 57 (3.3) | <.001 |

Data are presented as n (%). Abbreviations. CVC, central venous catheter; PVC, peripheral venous catheter; SSTI, skin and soft tissue infection; UTI, urinary tract infection; SSI, surgical site infection; IE, infective endocarditis; AVG, arteriovenous graft.

**Supplementary Table 3. Univariate logistic regression analysis of risk factors associated with metastatic infection in patients with *S. aureus* bacteremia**

| **Variable** | **OR** | **95% CI** | ***P* value** |
| --- | --- | --- | --- |
| Age > 60 y | 1.01 | 0.79 to 1.31 | 0.91 |
| Male | 1.06 | 0.81 to 1.38 | 0.68 |
| Methicillin–resistant *Staphylococcus aureus* | 0.86 | 0.67 to 1.10 | 0.23 |
| Mode of acquisition |  |  |  |
| Community-acquired | (reference) |  |  |
| Health-care associated | 0.42 | 0.30 to 0.60 | <0.001 |
| Nosocomial | 0.27 | 0.19 to 0.38 | <0.001 |
| Malignancy | 0.61 | 0.47 to 0.79 | <0.001 |
| Diabetes | 0.97 | 0.73 to 1.27 | 0.82 |
| Hypertension | 0.89 | 0.69 to 1.15 | 0.39 |
| End-stage renal disease | 1.25 | 0.84 to 1.82 | 0.26 |
| Liver cirrhosis | 0.83 | 0.57 to 1.18 | 0.31 |
| Immunosuppressant agent | 0.78 | 0.44 to 1.31 | 0.38 |
| Corticosteroid use | 0.63 | 0.46 to 0.86 | 0.01 |
| Central venous catheter | 0.69 | 0.53 to 0.90 | 0.01 |
| Cardiac implantable device | 0.78 | 0.18 to 2.31 | 0.69 |
| Prosthetic heart valve | 2.2 | 1.22 to 3.83 | 0.01 |
| Vascular graft | 1.36 | 0.86 to 2.08 | 0.17 |
| Orthopedic implant | 1.70 | 0.94 to 2.92 | 0.07 |
| Fever > 72 h | 2.13 | 1.64 to 2.77 | <0.001 |
| Persistent bacteremia (≥3 d) | 5.21 | 3.98 to 6.85 | <0.001 |
| Severity of infection |  |  |  |
| No sepsis | (reference) |  |  |
| Sepsis | 1.09 | 0.78 to 1.55 | 0.63 |
| Septic shock | 1.82 | 1.15 to 2.86 | 0.01 |
| Underlying medical conditions |  |  |  |
| Central venous catheter | 0.92 | 0.69 to 1.22 | 0.57 |
| Peripheral venous catheter | 0.49 | 0.24 to 0.88 | 0.03 |
| Pneumonia | 0.12 | 0.04 to 0.30 | <0.001 |
| Skin and soft tissue infection | 1.16 | 0.76 to 1.73 | 0.49 |
| Urinary tract infection | 0.22 | 0.01 to 1.07 | 0.14 |
| Surgical wound infection | 0.75 | 0.39 to 1.31 | 0.34 |
| Endocarditis | 16.20 | 9.34 to 29.50 | <0.001 |
| Bone | 2.09 | 1.31 to 3.27 | 0.002 |
| Joint | 1.65 | 0.86 to 2.98 | 0.11 |
| Arteriovenous graft infection | 2.57 | 1.36 to 4.68 | 0.003 |
| Others | 0.44 | 0.25 to 0.74 | 0.003 |
| Unknown primary focus | 0.73 | 0.50 to 1.05 | 0.10 |
| C-reactive protein ≥ 10 mg/dL | 2.83 | 2.17 to 3.71 | <0.001 |
| Time to appropriate antibiotic therapy, d | 0.99 | 0.90 to 1.07 | 0.76 |
| Duration of antibiotic therapy |  |  |  |
| 0–2 weeks | (reference) |  |  |
| >2 to ≤6 weeks | 1.09 | 0.74 to 1.65 | 0.68 |
| >6 weeks | 3.76 | 2.50 to 5.79 | <0.001 |
| Focus removal |  |  |  |
| Complete | (reference) |  |  |
| Not complete | 1.19 | 0.67 to 2.01 | 0.54 |
| No eradicable focus | 0.92 | 0.70 to 1.19 | 0.51 |

**Supplementary Table 4. Distribution of metastatic infection sites by primary infection focus within 90 days post–*Staphylococcus aureus* bacteremia**

| **Site of metastatic infection** | **CVC  (n=90)** | **PVC  (n=14)** | **Lung (n=4)** | **SSTI  (n=46)** | **UTI  (n=1)** | **SSI  (n=17)** | **IE  (n=105)** | **Bone (n=37)** | **Joint  (n=26)** | **AVG infection  (n=22)** | **Others  (n=23)** | **Unknown  (n=54)** | **Total  (n=439)** | ***P* value** |
| --- | --- | --- | --- | --- | --- | --- | --- | --- | --- | --- | --- | --- | --- | --- |
| CNS | 4 (4.4) | 0 (0.0) | 2 (50.0) | 0 (0.0) | 0 (0.0) | 5 (29.4) | 30 (28.6) | 8 (21.6) | 3 (11.5) | 0 (0.0) | 0 (0.0) | 2 (3.7) | 54 (12.3) | **<.001** |
| Heart valve | 12 (13.3) | 1 (7.1) | 0 (0.0) | 2 (4.3) | 0 (0.0) | 2 (11.8) | 0 (0.0) | 0 (0.0) | 0 (0.0) | 2 (9.1) | 1 (4.3) | 2 (3.7) | 22 (5.0) | 0.086 |
| Skin | 2 (2.2) | 0 (0.0) | 0 (0.0) | 1 (2.2) | 0 (0.0) | 0 (0.0) | 3 (2.9) | 0 (0.0) | 0 (0.0) | 1 (4.5) | 1 (4.3) | 1 (1.9) | 9 (2.1) | 0.924 |
| Soft tissue | 8 (8.9) | 3 (21.4) | 0 (0.0) | 8 (17.4) | 0 (0.0) | 2 (11.8) | 8 (7.6) | 8 (21.6) | 4 (15.4) | 1 (4.5) | 1 (4.3) | 8 (14.8) | 51 (11.6) | 0.353 |
| Bone | 11 (12.2) | 1 (7.1) | 1 (25.0) | 12 (26.1) | 0 (0.0) | 3 (17.6) | 9 (8.6) | 5 (13.5) | 7 (26.9) | 1 (4.5) | 4 (17.4) | 9 (16.7) | 63 (14.4) | 0.078 |
| Joint | 5 (5.6) | 0 (0.0) | 0 (0.0) | 9 (19.6) | 0 (0.0) | 1 (5.9) | 7 (6.7) | 4 (10.8) | 6 (23.1) | 3 (13.6) | 2 (8.7) | 6 (11.1) | 43 (9.8) | **0.034** |
| Kidney | 1 (1.1) | 1 (7.1) | 0 (0.0) | 0 (0.0) | 0 (0.0) | 0 (0.0) | 11 (10.5) | 1 (2.7) | 0 (0.0) | 0 (0.0) | 0 (0.0) | 6 (11.1) | 20 (4.6) | **<.001** |
| Eye | 12 (13.3) | 3 (21.4) | 1 (25.0) | 4 (8.7) | 0 (0.0) | 1 (5.9) | 13 (12.4) | 3 (8.1) | 1 (3.8) | 5 (22.7) | 8 (34.8) | 6 (11.1) | 57 (13.0) | **0.042** |
| Lung | 33 (36.7) | 4 (28.6) | 0 (0.0) | 8 (17.4) | 1 (100.0) | 3 (17.6) | 13 (12.4) | 5 (13.5) | 4 (15.4) | 9 (40.9) | 3 (13.0) | 12 (22.2) | 95 (21.6) | 0.061 |
| Others*** | 2 (2.2) | 1 (7.1) | 0 (0.0) | 2 (4.3) | 0 (0.0) | 0 (0.0) | 11 (10.5) | 3 (8.1) | 1 (3.8) | 0 (0.0) | 3 (13.0) | 2 (3.7) | 25 (5.7) | **0.02** |

Data are presented as n (%). Abbreviations. CVC, central venous catheter; PVC, peripheral venous catheter; SSTI, skin and soft tissue infection; UTI, urinary tract infection; SSI, surgical site infection; IE, infective endocarditis; AVG, arteriovenous graft. *P* values <.05 are in bold to denote statistical significance. “***Others” refers to metastatic infections occurring in the intra-abdominal region, vascular grafts, or spleen.

**Supplementary Table 5. Detailed symptom profile of ocular involvement in patients with *S. aureus* bacteremia**

|  | Patients (n=57) |
| --- | --- |
| Eye symptoms present | 22 (38.6) |
| Decreased visual acuity | 9/22 (40.9) |
| Visual disturbance | 9/22 (40.9) |
| Ocular pain | 5/22 (22.7) |
| Conjunctival injection or erythema | 4/22 (18.2) |
| No eye symptoms reported | 23 (40.4) |
| Symptom assessment not possible | 12 (21.1) |

Data are presented as n (%).

**Supplementary Table 6. Distribution of early metastatic infection sites (within 7 days post-bacteremia) by primary infection focus**

| Site of metastatic infection | CVC (n=79) | PVC (n=12) | Lung (n=3) | SSTI (n=35) | UTI  (n=1) | SSI (n=14) | IE (n=93) | Bone (n=29) | Joint (n=19) | AVG infection (n=18) | Others (n=19) | Unknown (n=38) | Total (n=363) | P value |
| --- | --- | --- | --- | --- | --- | --- | --- | --- | --- | --- | --- | --- | --- | --- |
| CNS | 4 (5.1) | 0 (0.0) | 1 (33.3) | 0 (0.0) | 0 (0.0) | 5 (35.7) | 30 (32.3) | 6 (20.7) | 2 (10.5) | 0 (0.0) | 0 (0.0) | 2 (5.3) | 50 (13.9) | **<.001** |
| Heart valve | 11 (13.9) | 0 (0.0) | 0 (0.0) | 1 (2.9) | 0 (0.0) | 1 (7.1) | 0 (0.0) | 0 (0.0) | 0 (0.0) | 2 (11.1) | 1 (5.3) | 1 (2.6) | 17 (4.7) | 0.062 |
| Skin | 1 (1.3) | 0 (0.0) | 0 (0.0) | 1 (2.9) | 0 (0.0) | 0 (0.0) | 3 (3.2) | 0 (0.0) | 0 (0.0) | 1 (5.6) | 1 (5.3) | 1 (2.6) | 8 (2.2) | 0.888 |
| Soft tissue | 7 (8.9) | 3 (25.0) | 0 (0.0) | 8 (22.9) | 0 (0.0) | 2 (14.3) | 7 (7.5) | 8 (27.6) | 4 (21.1) | 1 (5.6) | 1 (5.3) | 7 (18.4) | 48 (13.3) | 0.107 |
| Bone | 7 (8.9) | 0 (0.0) | 1 (33.3) | 7 (20.0) | 0 (0.0) | 2 (14.3) | 5 (5.4) | 3 (10.3) | 4 (21.1) | 1 (5.6) | 2 (10.5) | 3 (7.9) | 36 (9.7) | 0.297 |
| Joint | 3 (3.8) | 0 (0.0) | 0 (0.0) | 6 (17.1) | 0 (0.0) | 1 (7.1) | 4 (4.3) | 2 (6.9) | 4 (21.1) | 1 (5.6) | 0 (0.0) | 2 (5.3) | 25 (6.4) | **0.042** |
| Kidney | 1 (1.3) | 1 (8.3) | 0 (0.0) | 0 (0.0) | 0 (0.0) | 0 (0.0) | 11 (11.8) | 1 (3.4) | 0 (0.0) | 0 (0.0) | 0 (0.0) | 6 (15.8) | 20 (5.6) | **<.001** |
| Eye | 10 (12.7) | 3 (25.0) | 1 (33.3) | 3 (8.6) | 0 (0.0) | 0 (0.0) | 9 (9.7) | 2 (6.9) | 0 (0.0) | 4 (22.2) | 8 (42.1) | 6 (15.8) | 46 (12.8) | 0.009 |
| Lung | 33 (41.8) | 4 (33.3) | 0 (0.0) | 7 (20.0) | 1 (100.0) | 3 (21.4) | 13 (14.0) | 4 (13.8) | 4 (21.1) | 8 (44.4) | 3 (15.8) | 9 (23.7) | 89 (24.7) | 0.072 |
| Others*** | 2 (2.5) | 1 (8.3) | 0 (0.0) | 2 (5.7) | 0 (0.0) | 0 (0.0) | 11 (11.8) | 3 (10.3) | 1 (5.3) | 0 (0.0) | 3 (15.8) | 1 (2.6) | 24 (6.7) | **0.032** |

Data are presented as n (%). Abbreviations. CVC, central venous catheter; PVC, peripheral venous catheter; SSTI, skin and soft tissue infection; UTI, urinary tract infection; SSI, surgical site infection; IE, infective endocarditis; AVG, arteriovenous graft. *P* values <.05 are in bold to denote statistical significance. *“**Others” refers to metastatic infections occurring in the intra-abdominal region, vascular grafts, or spleen.

**Supplementary Table 7.** **Distribution of late metastatic infection sites (beyond 7 days post-bacteremia) by primary infection focus**

| Site of metastatic infection | CVC (n=10) | PVC (n=2) | Lung (n=1) | SSTI (n=10) | UTI (n=0) | SSI (n=3) | IE (n=11) | Bone (n=8) | Joint (n=7) | AVG infection (n=3) | Others (n=2) | Unknown (n=16) | Total (n=76) | P value |
| --- | --- | --- | --- | --- | --- | --- | --- | --- | --- | --- | --- | --- | --- | --- |
| CNS | 0 (0.0) | 0 (0.0) | 1 (100.0) | 0 (0.0) | *NA* | 0 (0.0) | 0 (0.0) | 2 (25.0) | 1 (14.3) | 0 (0.0) | 0 (0.0) | 0 (0.0) | 4 (5.5) | **0.004** |
| Heart valve | 1 (10.0) | 1 (50.0) | 0 (0.0) | 1 (10.0) | *NA* | 1 (33.3) | 0 (0.0) | 0 (0.0) | 0 (0.0) | 0 (0.0) | 0 (0.0) | 1 (6.2) | 5 (6.8) | 0.514 |
| Skin | 1 (10.0) | 0 (0.0) | 0 (0.0) | 0 (0.0) | *NA* | 0 (0.0) | 0 (0.0) | 0 (0.0) | 0 (0.0) | 0 (0.0) | 0 (0.0) | 0 (0.0) | 1 (1.4) | 0.795 |
| Soft tissue | 1 (10.0) | 0 (0.0) | 0 (0.0) | 0 (0.0) | *NA* | 0 (0.0) | 1 (9.1) | 0 (0.0) | 0 (0.0) | 0 (0.0) | 0 (0.0) | 1 (6.2) | 3 (4.1) | 0.971 |
| Bone | 4 (40.0) | 1 (50.0) | 0 (0.0) | 4 (40.0) | *NA* | 1 (33.3) | 4 (36.4) | 2 (25.0) | 3 (42.9) | 0 (0.0) | 1 (50.0) | 6 (37.5) | 27 (35.6) | 0.867 |
| Joint | 1 (10.0) | 0 (0.0) | 0 (0.0) | 3 (30.0) | *NA* | 0 (0.0) | 2 (18.2) | 2 (25.0) | 2 (28.6) | 1 (33.3) | 1 (50.0) | 4 (25.0) | 18 (21.9) | 0.705 |
| Kidney | 0 (0.0) | 0 (0.0) | 0 (0.0) | 0 (0.0) | *NA* | 0 (0.0) | 0 (0.0) | 0 (0.0) | 0 (0.0) | 0 (0.0) | 0 (0.0) | 0 (0.0) | 0 (0.0) | *NA* |
| Eye | 2 (20.0) | 0 (0.0) | 0 (0.0) | 1 (10.0) | *NA* | 1 (33.3) | 4 (36.4) | 1 (12.5) | 1 (14.3) | 1 (33.3) | 0 (0.0) | 0 (0.0) | 11 (15.1) | 0.587 |
| Lung | 0 (0.0) | 0 (0.0) | 0 (0.0) | 1 (10.0) | *NA* | 0 (0.0) | 0 (0.0) | 1 (12.5) | 0 (0.0) | 1 (33.3) | 0 (0.0) | 3 (18.8) | 6 (8.2) | 0.535 |
| Others*** | 0 (0.0) | 0 (0.0) | 0 (0.0) | 0 (0.0) | *NA* | 0 (0.0) | 0 (0.0) | 0 (0.0) | 0 (0.0) | 0 (0.0) | 0 (0.0) | 1 (6.2) | 1 (1.4) | 0.935 |

Data are presented as n (%). Abbreviations. CVC, central venous catheter; PVC, peripheral venous catheter; SSTI, skin and soft tissue infection; UTI, urinary tract infection; SSI, surgical site infection; IE, infective endocarditis; BJI, bone and joint infection; AVG, arteriovenous graft; NA, not available. *P* values <.05 are in bold to denote statistical significance. *“**Others” refers to metastatic infections occurring in the intra-abdominal region, vascular grafts, or spleen.

**Supplementary Table 8. Metastatic infection site distribution in patients with MSSA bacteremia**

| Site of metastatic infection | AVG infection (N=8) | Bone (N=22) | CVC (N=19) | IE (N=71) | Joint (N=9) | Others (N=14) | Lung (N=1) | PVC (N=10) | SSTI (N=29) | SSI (N=6) | Unknown (N=37) | UTI (N=1) | Total (N=227) | P |
| --- | --- | --- | --- | --- | --- | --- | --- | --- | --- | --- | --- | --- | --- | --- |
| CNS | 0 (0.0%) | 5 (22.7%) | 2 (10.5%) | 22 (31.0%) | 0 (0.0%) | 0 (0.0%) | 0 (0.0%) | 0 (0.0%) | 0 (0.0%) | 1 (16.7%) | 1 (2.7%) | 0 (0.0%) | 31 (13.7%) | **<0.001** |
| Heart valve | 1 (12.5%) | 0 (0.0%) | 2 (10.5%) | 0 (0.0%) | 0 (0.0%) | 1 (7.1%) | 0 (0.0%) | 0 (0.0%) | 0 (0.0%) | 1 (16.7%) | 2 (5.4%) | 0 (0.0%) | 7 (3.1%) | 0.361 |
| Skin | 1 (12.5%) | 0 (0.0%) | 0 (0.0%) | 1 (1.4%) | 0 (0.0%) | 1 (7.1%) | 0 (0.0%) | 0 (0.0%) | 0 (0.0%) | 0 (0.0%) | 1 (2.7%) | 0 (0.0%) | 4 (1.8%) | 0.630 |
| Soft tissue | 0 (0.0%) | 4 (18.2%) | 1 (5.3%) | 6 (8.5%) | 3 (33.3%) | 1 (7.1%) | 0 (0.0%) | 2 (20.0%) | 5 (17.2%) | 1 (16.7%) | 4 (10.8%) | 0 (0.0%) | 27 (11.9%) | 0.613 |
| Bone | 1 (12.5%) | 2 (9.1%) | 3 (15.8%) | 6 (8.5%) | 2 (22.2%) | 2 (14.3%) | 1 (100.0%) | 1 (10.0%) | 8 (27.6%) | 1 (16.7%) | 5 (13.5%) | 0 (0.0%) | 32 (14.1%) | 0.542 |
| Joint | 0 (0.0%) | 2 (9.1%) | 0 (0.0%) | 4 (5.6%) | 2 (22.2%) | 1 (7.1%) | 0 (0.0%) | 0 (0.0%) | 6 (20.7%) | 0 (0.0%) | 4 (10.8%) | 0 (0.0%) | 19 (8.4%) | 0.294 |
| Kidney | 0 (0.0%) | 1 (4.5%) | 1 (5.3%) | 6 (8.5%) | 0 (0.0%) | 0 (0.0%) | 0 (0.0%) | 1 (10.0%) | 0 (0.0%) | 0 (0.0%) | 5 (13.5%) | 0 (0.0%) | 14 (6.2%) | 0.327 |
| Eye | 1 (12.5%) | 3 (13.6%) | 2 (10.5%) | 10 (14.1%) | 0 (0.0%) | 5 (35.7%) | 0 (0.0%) | 3 (30.0%) | 4 (13.8%) | 1 (16.7%) | 6 (16.2%) | 0 (0.0%) | 35 (15.4%) | 0.482 |
| Lung | 4 (50.0%) | 4 (18.2%) | 8 (42.1%) | 7 (9.9%) | 1 (11.1%) | 1 (7.1%) | 0 (0.0%) | 2 (20.0%) | 4 (13.8%) | 1 (16.7%) | 7 (18.9%) | 1 (100.0%) | 40 (17.6%) | 0.197 |
| Others*** | 0 (0.0%) | 1 (4.5%) | 0 (0.0%) | 9 (12.7%) | 1 (11.1%) | 2 (14.3%) | 0 (0.0%) | 1 (10.0%) | 2 (6.9%) | 0 (0.0%) | 2 (5.4%) | 0 (0.0%) | 18 (7.9%) | 0.238 |

Data are presented as n (%). Abbreviations. CVC, central venous catheter; PVC, peripheral venous catheter; SSTI, skin and soft tissue infection; UTI, urinary tract infection; SSI, surgical site infection; IE, infective endocarditis; BJI, bone and joint infection; AVG, arteriovenous graft; NA, not available. *P* values <.05 are in bold to denote statistical significance. *“**Others” refers to metastatic infections occurring in the intra-abdominal region, vascular grafts, or spleen.

**Supplementary Table 9. Metastatic infection site distribution in patients with MRSA bacteremia**

| sSite of metastatic infection | AVG infection (N=14) | Bone (N=15) | CVC (N=71) | IE (N=34) | Joint  (N=17) | Others (N=9) | Lung (N=3) | PVC (N=4) | SSTI (N=17) | SSI (N=11) | Unknown (N=17) | UTI (N=0) | Total (N=212) | P |
| --- | --- | --- | --- | --- | --- | --- | --- | --- | --- | --- | --- | --- | --- | --- |
| CNS | 0 (0.0%) | 3 (20.0%) | 2 (2.8%) | 8 (23.5%) | 3 (17.6%) | 0 (0.0%) | 2 (66.7%) | 0 (0.0%) | 0 (0.0%) | 4 (36.4%) | 1 (5.9%) | 0 (0.0%) | 23 (10.8%) | **<0.001** |
| Heart valve | 1 (7.1%) | 0 (0.0%) | 10 (14.1%) | 0 (0.0%) | 0 (0.0%) | 0 (0.0%) | 0 (0.0%) | 1 (25.0%) | 2 (11.8%) | 1 (9.1%) | 0 (0.0%) | 0 (0.0%) | 15 (7.1%) | 0.306 |
| Skin | 0 (0.0%) | 0 (0.0%) | 2 (2.8%) | 2 (5.9%) | 0 (0.0%) | 0 (0.0%) | 0 (0.0%) | 0 (0.0%) | 1 (5.9%) | 0 (0.0%) | 0 (0.0%) | 0 (0.0%) | 5 (2.4%) | 0.683 |
| Soft tissue | 1 (7.1%) | 4 (26.7%) | 7 (9.9%) | 2 (5.9%) | 1 (5.9%) | 0 (0.0%) | 0 (0.0%) | 1 (25.0%) | 3 (17.6%) | 1 (9.1%) | 4 (23.5%) | 0 (0.0%) | 24 (11.3%) | 0.324 |
| Bone | 0 (0.0%) | 3 (20.0%) | 8 (11.3%) | 3 (8.8%) | 5 (29.4%) | 2 (22.2%) | 0 (0.0%) | 0 (0.0%) | 4 (23.5%) | 2 (18.2%) | 4 (23.5%) | 0 (0.0%) | 31 (14.6%) | **0.047** |
| Joint | 3 (21.4%) | 2 (13.3%) | 5 (7.0%) | 3 (8.8%) | 4 (23.5%) | 1 (11.1%) | 0 (0.0%) | 0 (0.0%) | 3 (17.6%) | 1 (9.1%) | 2 (11.8%) | 0 (0.0%) | 24 (11.3%) | 0.251 |
| Kidney | 0 (0.0%) | 0 (0.0%) | 0 (0.0%) | 5 (14.7%) | 0 (0.0%) | 0 (0.0%) | 0 (0.0%) | 0 (0.0%) | 0 (0.0%) | 0 (0.0%) | 1 (5.9%) | 0 (0.0%) | 6 (2.8%) | **<0.001** |
| Eye | 4 (28.6%) | 0 (0.0%) | 10 (14.1%) | 3 (8.8%) | 1 (5.9%) | 3 (33.3%) | 1 (33.3%) | 0 (0.0%) | 0 (0.0%) | 0 (0.0%) | 0 (0.0%) | 0 (0.0%) | 22 (10.4%) | **0.023** |
| Lung | 5 (35.7%) | 1 (6.7%) | 25 (35.2%) | 6 (17.6%) | 3 (17.6%) | 2 (22.2%) | 0 (0.0%) | 2 (50.0%) | 4 (23.5%) | 2 (18.2%) | 5 (29.4%) | 0 (0.0%) | 55 (25.9%) | 0.581 |
| Others*** | 0 (0.0%) | 2 (13.3%) | 2 (2.8%) | 2 (5.9%) | 0 (0.0%) | 1 (11.1%) | 0 (0.0%) | 0 (0.0%) | 0 (0.0%) | 0 (0.0%) | 0 (0.0%) | 0 (0.0%) | 7 (3.3%) | 0.269 |

Data are presented as n (%). Abbreviations. CVC, central venous catheter; PVC, peripheral venous catheter; SSTI, skin and soft tissue infection; UTI, urinary tract infection; SSI, surgical site infection; IE, infective endocarditis; BJI, bone and joint infection; AVG, arteriovenous graft; NA, not available. *P* values <.05 are in bold to denote statistical significance. *“**Others” refers to metastatic infections occurring in the intra-abdominal region, vascular grafts, or spleen.

**Supplementary Table 10. Multivariable logistic regression analysis for the association between clinical outcomes and metastatic infections**

|  | **90-day mortality** | | **90-day recurrence** | |
| --- | --- | --- | --- | --- |
| **Variables** | **OR (95% CI)** | ***P* value** | **OR (95% CI)** | ***P* value** |
| Metastatic infection site |  |  |  |  |
| Central nervous system | 2.59 (1.24-5.37) | **.010** | 0.51 (0.07-2.15) | .419 |
| Heart valve | 2.91 (1.15-7.33) | **.022** | *NA* |  |
| Skin | 0.30 (0.02-1.98) | .290 | *NA* |  |
| Soft tissue | 0.41 (0.16-0.94) | **.048** | 1.21 (0.28-3.65) | .760 |
| Bone and joint | 0.65 (0.32-1.23) | .207 | 1.85 (0.67-4.35) | .191 |
| Kidney | 0.44 (0.09-1.52) | .233 | *NA* |  |
| Eye | 0.76 (0.37-1.47) | .424 | 1.08 (0.25-3.21) | .898 |
| Lung | 1.83 (1.11-2.98) | **.017** | 0.69 (0.20-1.80) | .502 |
| Others* | 1.45 (0.55-3.64) | .440 | 1.12 (0.06-6.10) | .916 |

All of the results in the multivariable analyses were adjusted for age, sex, underlying diseases, severity of infection, and primary infection focus of *S. aureus* bacteremia. Abbreviations. OR, odds ratio; CI, confidence interval. *P* values <.05 are in bold to denote statistical significance. *“**Others” refers to metastatic infections occurring in the intra-abdominal region, vascular grafts, or spleen.


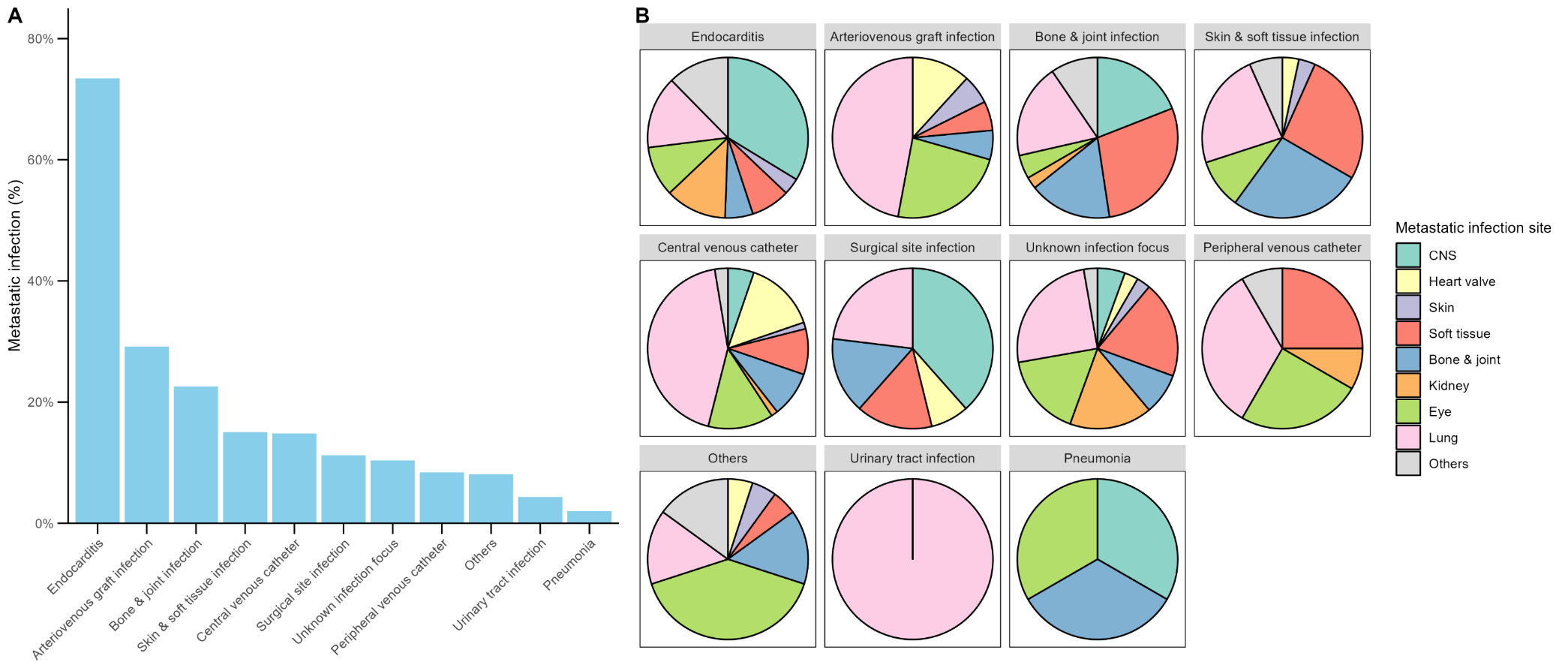


**Supplementary Figure 1. Early metastatic infection profiles by primary infection focus in *Staphylococcus aureus* bacteremia.** *A*. Risk of early metastatic infections based on *S. aureus* bacteremia infection foci. *B*. Pie charts showing the proportional distributions of early metastatic infection sites, categorized by primary infection focus and arranged in descending order of infection risk.


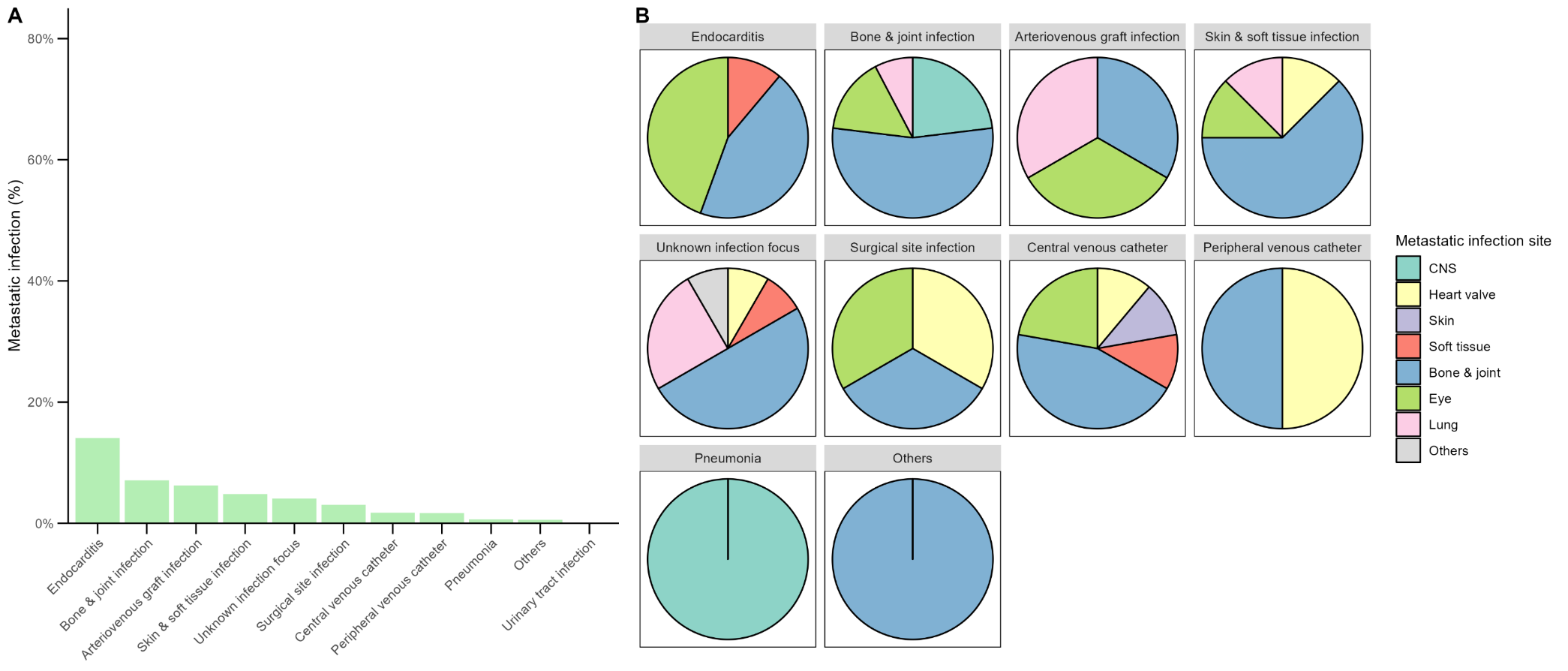


**Supplementary Figure 2. Late metastatic infection profiles by primary infection focus in *Staphylococcus aureus* bacteremia.** *A*. Risk of late metastatic infections associated with *S. aureus* bacteremia infection foci. *B*. Pie charts illustrating the proportional distributions of late metastatic infection sites, categorized by primary infection focus and arranged according to descending metastatic infection risk.
